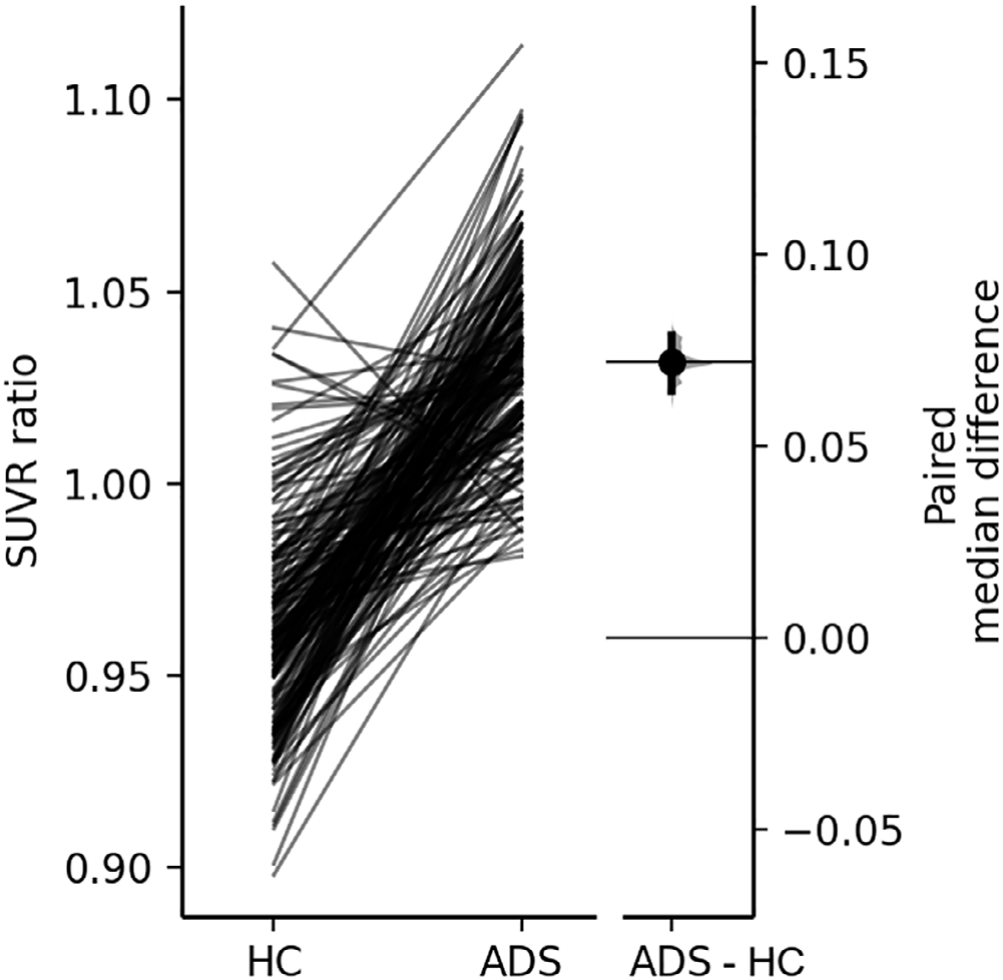# Microglial Activation and Tau Accumulation in the Alzheimer's Disease Spectrum: Insights from Longitudinal PET Imaging

**DOI:** 10.1002/alz70856_101228

**Published:** 2025-12-24

**Authors:** Marco Öchsner, Matthias Brendel, Nicolai Franzmeier, Lena‐Katharina Trappmann, Mirlind Zaganjori, Ersin Ersözlü, Estrella Morenas‐Rodriguez, Selim Üstün Guersel, Lena Burow, Carolin Isabella Kurz, Jan Haeckert, Maia Tato, Julia Utecht, Boris Papazov, Oliver Michael Pogarell, Daniel Janowitz, Katharina Buerger, Michael Ewers, Carla Palleis, Endy Weidinger, Gloria Biechele, Sebastian Schuster, Anika Finze, Florian Eckenweber, Rainer Rupprecht, Axel Rominger, Oliver Goldhardt, Timo Grimmer, Daniel Keeser, Sophia Stöcklein, Olaf Dietrich, Peter Bartenstein, Johannes Levin, Günter U Höglinger, Robert Perneczky, Boris‐Stephan Rauchmann

**Affiliations:** ^1^ LMU, München, Bavaria, Germany; ^2^ Department of Nuclear Medicine, University Hospital, LMU Munich, Munich, Bavaria, Germany; ^3^ Institute for Stroke and Dementia Research (ISD), LMU University Hospital, LMU, Munich, Bavaria, Germany; ^4^ University Hospital, LMU Munich, Munich, Germany; ^5^ LMU University Hospital, Munich, Germany; ^6^ Klinik für Psychiatrie und Psychotherapie, Charité, Berlin, Bavaria, Germany; ^7^ German Center for Neurodegenerative Diseases (DZNE) and Metabolic Biochemistry, Biomedical Center (BMC), Munich, Germany; ^8^ Department of Psychiatry and Psychotherapy, University Hospital, LMU Munich, Munich, Bavaria, Germany; ^9^ Department of Psychiatry and Psychotherapy, LMU Hospital, LMU Munich, Munich, Germany; ^10^ Institute for Stroke and Dementia Research (ISD), University Hospital, LMU, Munich, Germany; ^11^ Institute for Stroke and Dementia Research (ISD), LMU University Hospital, LMU Munich, Munich, Germany; ^12^ Department of Neurology, Klinikum der Ludwig‐Maximilians Universität München, Munich, Bavaria, Germany; ^13^ Department of Neurology, University Hospital, Ludwig‐Maximilians‐Universität, Munich, Bavaria, Germany; ^14^ University Hospital of Munich, Munich, Germany; ^15^ University Hospital, Ludwig‐Maxmilians‐Universität, Munich, Germany; ^16^ University of Regensburg, Regensburg, Germany; ^17^ Inselspital Bern, Bern, Switzerland; ^18^ Technical University of Munich, School of Medicine and Health, TUM University Hospital, Center for Cognitive Disorders, Munich, Bavaria, Germany; ^19^ Department of Radiology, University Hospital, LMU Munich, Munich, Bavaria, Germany; ^20^ University Hospital, LMU Munich, München, Germany; ^21^ Department of Nuclear Medicine, University Hospital, LMU, Munich, Germany; ^22^ Department of Neurology, LMU University Hospital, LMU Munich, Munich, Munich, Germany; ^23^ Department of Neurology, Klinikum der Ludwig‐Maximilians Universität München, Munich, Germany; ^24^ Department of Psychiatry and Psychotherapy, Klinikum der Ludwig‐Maximilians Universität München, Munich, Germany; ^25^ Sheffield Institute for Translational Neuroscience, University of Sheffield, Sheffield, United Kingdom; ^26^ Department of Psychiatry and Psychotherapy, University Hospital, LMU Munich, Munich, Germany; ^27^ German Center for Neurodegenerative Diseases (DZNE), Munich, Germany; ^28^ Department of Neuroradiology, LMU University Hospital, Munich, Germany, Munich, Germany

## Abstract

**Background:**

Recent evidence suggests that microglial activation mirrors tau accumulation along highly connected brain regions, but their relation remains unclear. We examined (a) longitudinal changes in microglial activation in Alzheimer's disease spectrum (ADS) compared to healthy controls (HC) (b) whether these changes are linked to tau levels and their functional connectivity (FC), and (c) the relation of microglia and tau across CDR‐based groups.

**Method:**

As part of the longitudinal ActiGliA prospective cohort study, ADS (*n* = 36, defined by CSF Aβ42/Aβ40 ratio or an Aβ PET composite of ADS) and HC (*n* = 20, with CDR=0 and no Aβ pathology) underwent [18F]GE‐180 (TSPO) imaging to assess microglial activation and resting‐state fMRI to determine FC, alongside structural T1 MRI. After 18 months, a subset of participants received follow‐up TAU‐PET ([18F]Flutemetamol) and TSPO‐PET.

**Results:**

TSPO ratios (FU/BL SUVRs) increased from baseline to follow‐up in ADS when compared to HC (paired median difference 0.0718, *p* <0.001), and were negatively correlated with TAU (⍴=‐0.207, *p* = 0.003) or TSPO SUVRs (⍴=‐0.152, *p* = 0.032), while HCs showed positive correlations. For ADS participants, the TSPO SUVR ratio (⍴=‐0.19, *p* = 0.008) and ADS‐HC TSPO ratio difference (⍴=‐0.27, *p* <0.001) were inversely associated with FC distance from the TAU hotspot, a pattern opposite in HCs. Braak‐like stage analysis showed TSPO SUVR increases in ADS at advanced stages (Braak 5: 0.011, Braak 6: 0.0115) but decreases in HC. TAU SUVRs in CDR=0 (*n* = 13) were better predicted by TSPO SUVRs in CDR=0.5 (*n* = 6) (β=1.16, R^2^=0.79, AIC=‐509) than CDR=0 TSPO SUVRs (β=1.3, R^2^=0.64, AIC=‐307). Similarly, the TAU SUVRs in CDR=0.5 were better estimated by CDR=0.5 TSPO SUVRs (β=0.79, R2=0.64, AIC=‐400), than in CDR=0 (β=0.89, R2=0.5, AIC=‐239).

**Conclusion:**

These findings suggest microglial activation increases in ADS and correlates inversely with TAU, with spatial and temporal patterns supporting a mild pseudotemporal precedence of microglial activation over tau accumulation.